# Supplementary material for: Epigenetically reprogrammed methylation landscape drives the DNA self-assembly and serves as a universal cancer biomarker
Source: Nat Commun. 2018 Dec 4;9:4915. doi: 10.1038/s41467-018-07214-w (PMC6279781; doi:10.1038/s41467-018-07214-w)
Supplement: Supplementary file 1 — Supplementary Information [file 41467_2018_7214_MOESM1_ESM.pdf]

**Supplementary Information**

**of**

# Epigenetically reprogrammed methylation landscape drives the DNA self-assembly and serves as a universal cancer biomarker

Abu Ali Ibn Sina†[1], Laura G. Carrascosa†*[1], Ziyu Liang,[1] Yadveer S. Grewal,[1] Andri Wardiana,[1] Muhammad J. A. Shiddiky,[1] , Frank Gardiner[4], Hemamali Samaratunga[4], Maher Gandhi[5], Rodney Scott[3], Darren Korbie,[1] and Matt Trau*[1,2]

[1]Center for Personalised Nanomedicine, Australian Institute for Bioengineering and Nanotechnology (AIBN), Corner of College and Cooper Roads (Bldg 75), The University of Queensland, Brisbane QLD 4072, Australia

[2]School of Chemistry and Molecular Biosciences, The University of Queensland, Brisbane, QLD 4072, Australia.

[3]School of Biomedical Science and Pharmacy, The University of Newcastle, Callaghan NSW 2308 Australia

[4]School of Medicine, The University of Queensland, Brisbane, QLD 4072, Australia

[5]Diamantina Institute, The University of Queensland, Brisbane, QLD 4072, Australia

Tel: +61-7-33464178; Fax: +61-7-33463973

*Corresponding authors

†Joint first-authors

## Supplementary Figures

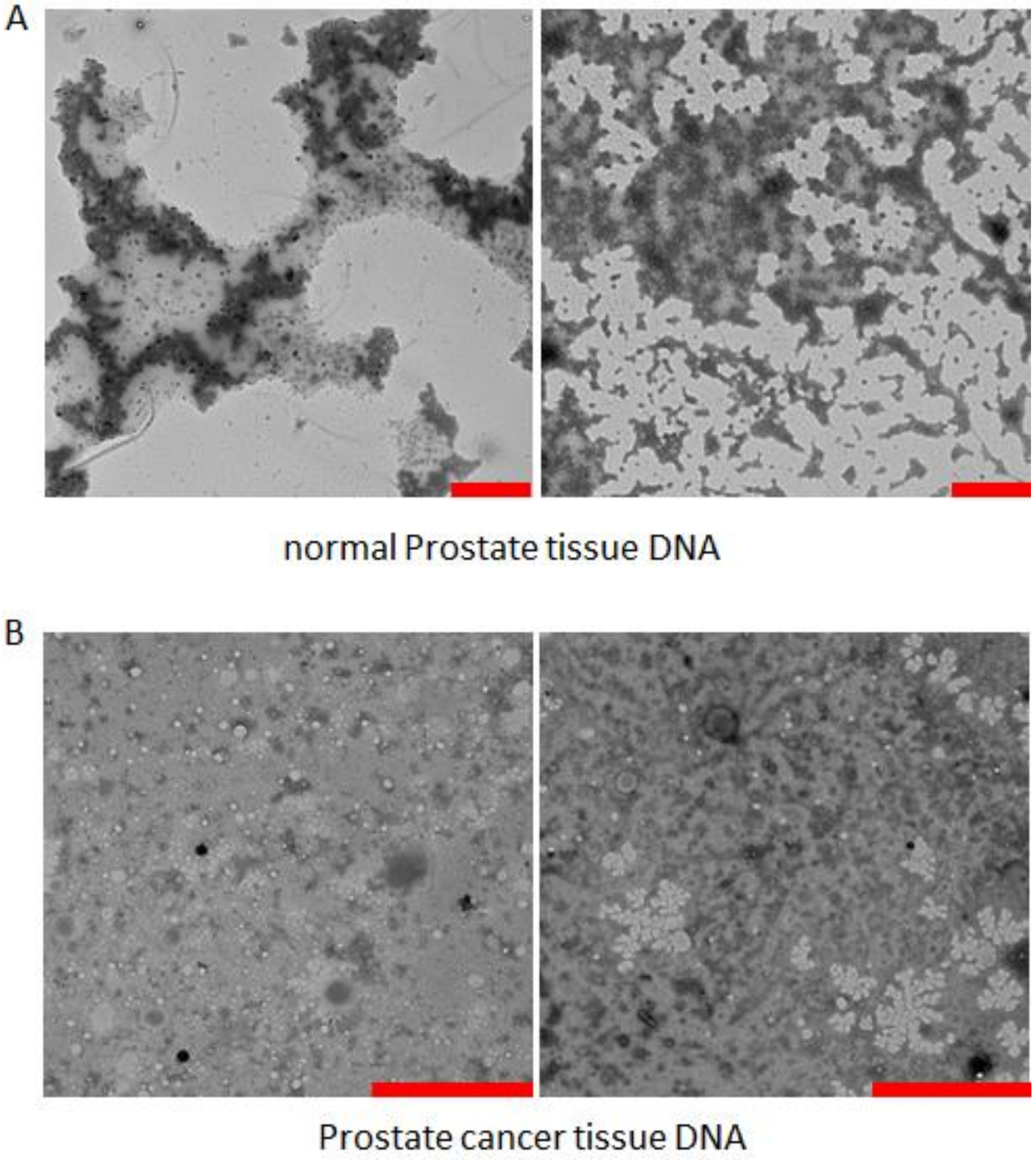


**Supplementary Figure 1.** Additional TEM image of gDNA. A) gDNA derived from normal prostate tissue and B) gDNA derived from prostate cancer tissue. Scale bars are 2000nm for all the supplementary figures.

A

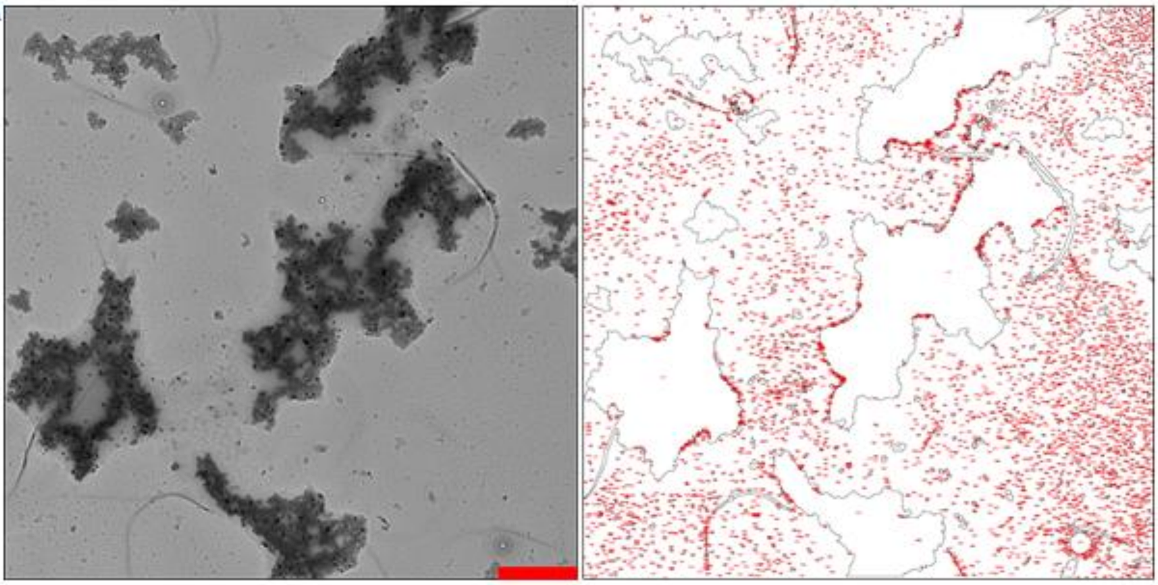

| Sample | Aggregates Count | Total Area ($nm^2$) | Average Size ($nm^2$) | %Area |
|---|---|---|---|---|
| gDNA (Normal Prostate tissue) | 5464 | 45341257.06 | 8298.18 | 25.369 |

B

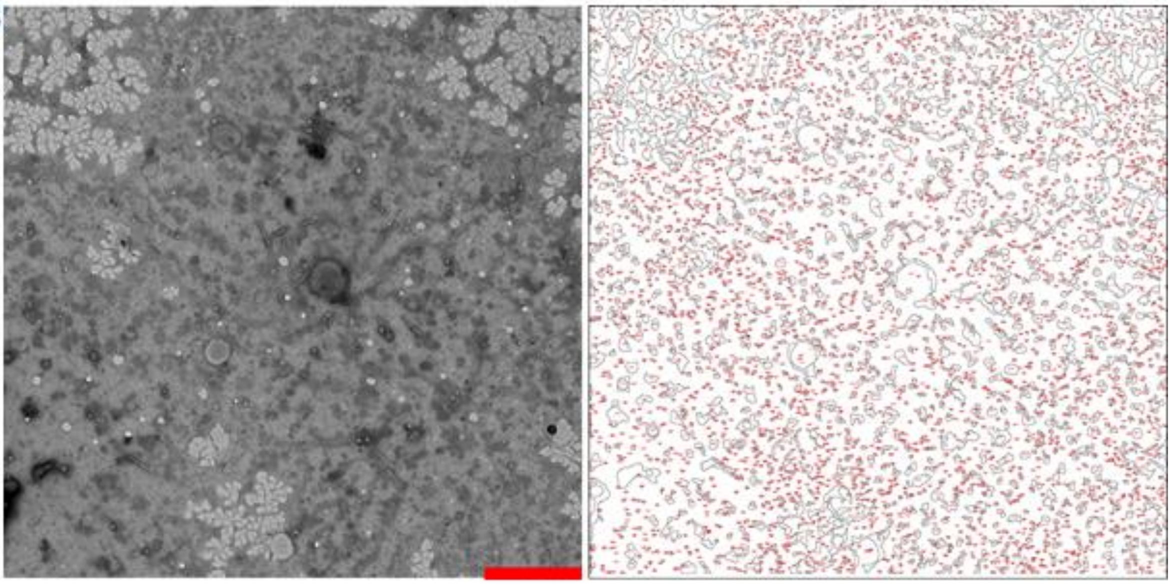

| Sample | Aggregates Count | Total Area ($nm^2$) | Average Size ($nm^2$) | %Area |
|---|---|---|---|---|
| gDNA (Cancer Prostate tissue) | 3933 | 6057435.111 | 1540.156 | 14.608 |

**Supplementary Figure 2.** ImageJ analysis for the TEM image of gDNA A) gDNA from normal tissue and B) gDNA from cancer tissue. Scale bars are 2000nm for all the supplementary figures.

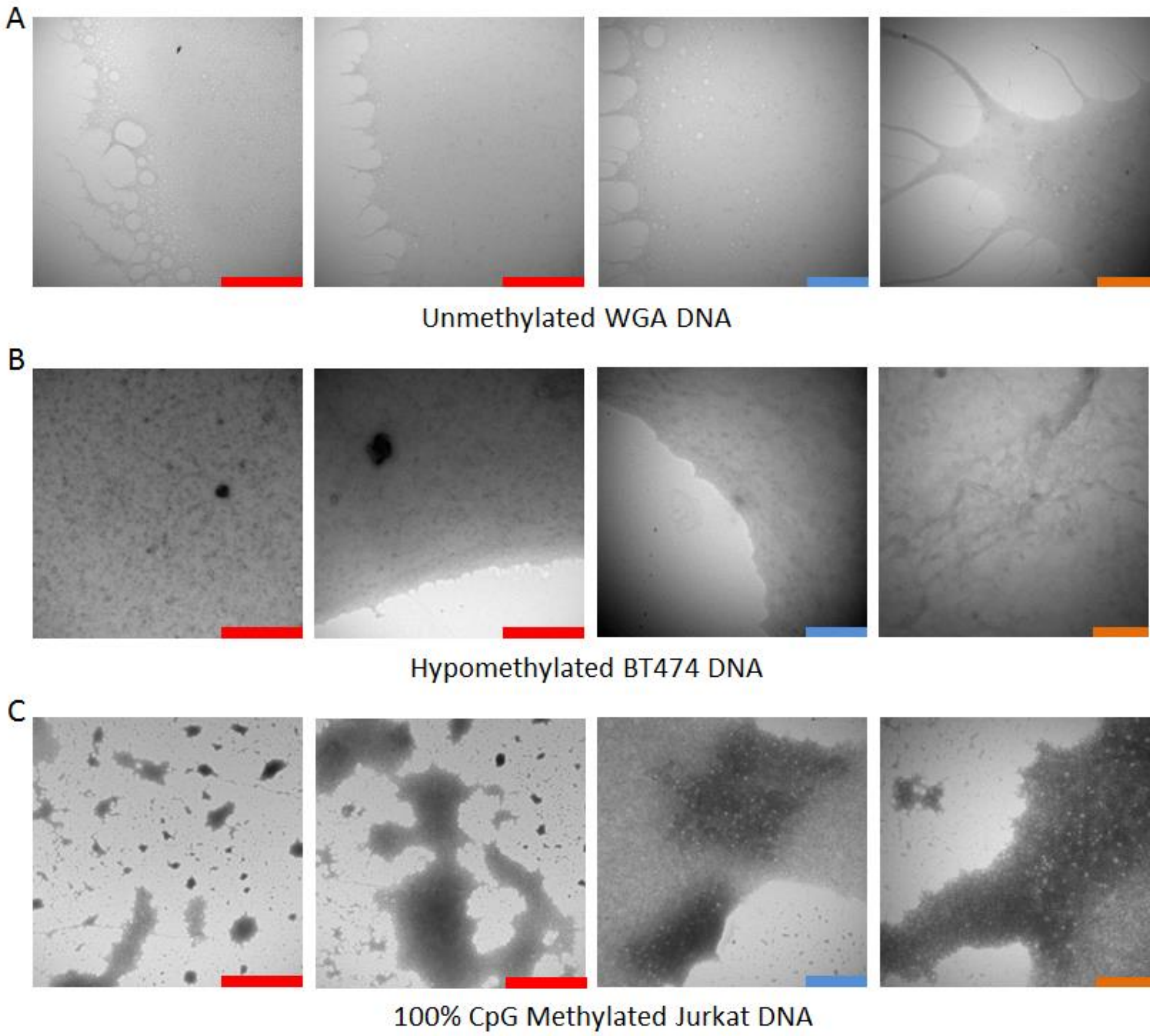


**Supplementary Figure 3.** Additional TEM images of gDNA with different methylation status. A) Unmethylated WGA DNA B) Hypomethylated BT474 breast cancer cell line DNA (43% global methylation) C) 100% Methylated Jurkat DNA. Scale bars are 2000nm (red) 1000nm (blue) and 500nm (orange) for all the supplementary figures.

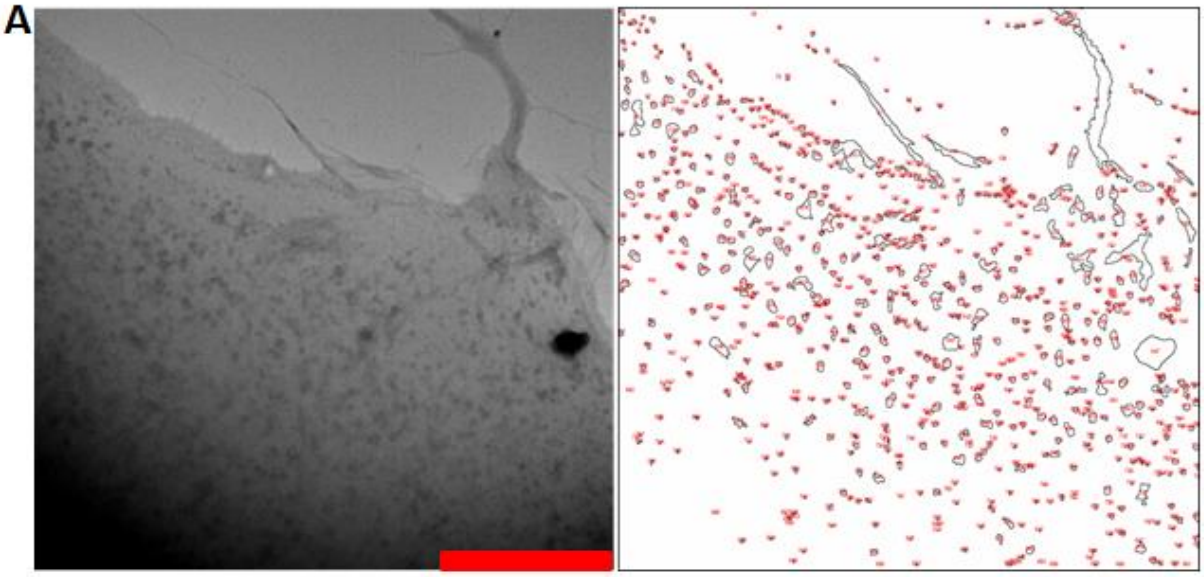


| Sample | Aggregates Count | Total Area ($nm^2$) | Average Size ($nm^2$) | %Area |
|---|---|---|---|---|
| gDNA (BT474 Breast Cancer Cell Line) | 781 | 2461565.146 | 3151.812 | 4.734 |

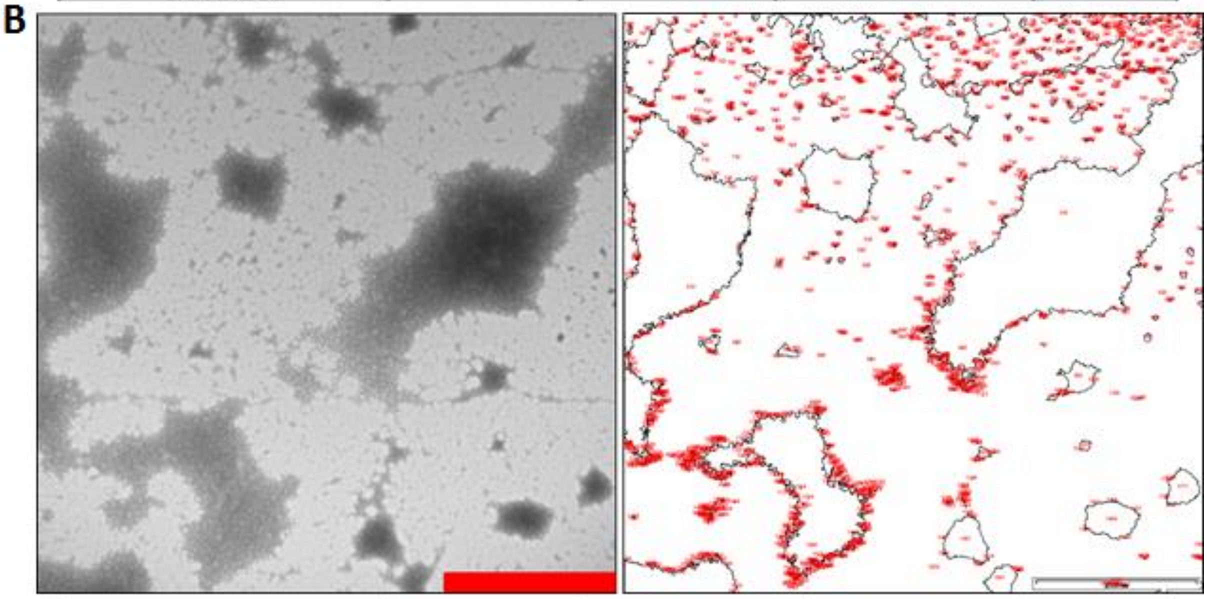


| Sample | Aggregates Count | Total Area ($nm^2$) | Average Size ($nm^2$) | %Area |
|---|---|---|---|---|
| gDNA (100% CpG Methylated Jurkat) | 1623 | 13501773.07 | 8319.02 | 25.99 |

**Supplementary Figure 4**. ImageJ analysis for the TEM image of DNA with different methylation status. A) gDNA from BT474 breast cancer cell line B) 100% CpG methylated Jurkat DNA. Scale bars are 2000nm for all the supplementary figures.

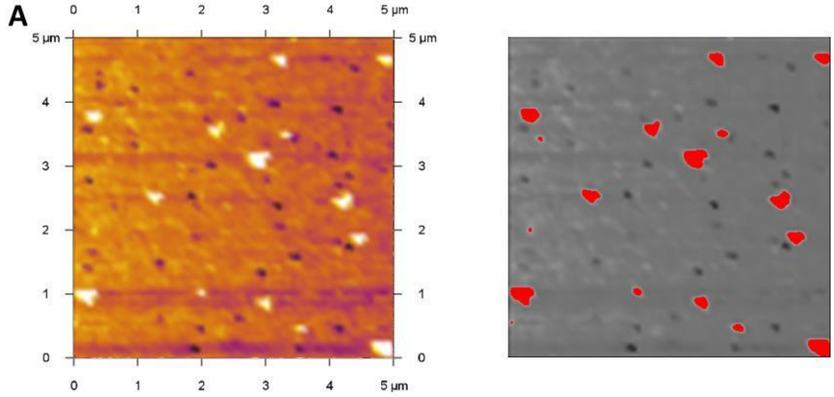


| Sample | Area Coverage |
|---|---|
| WGA | 2.35% |

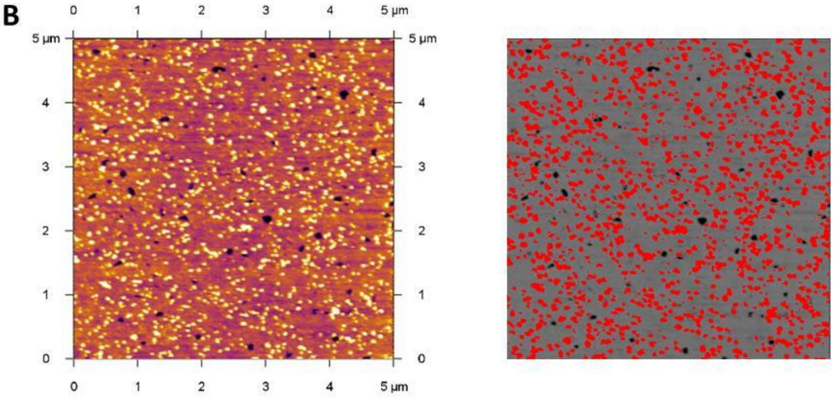


| Sample | Area Coverage |
|---|---|
| BT474 | 21.24% |

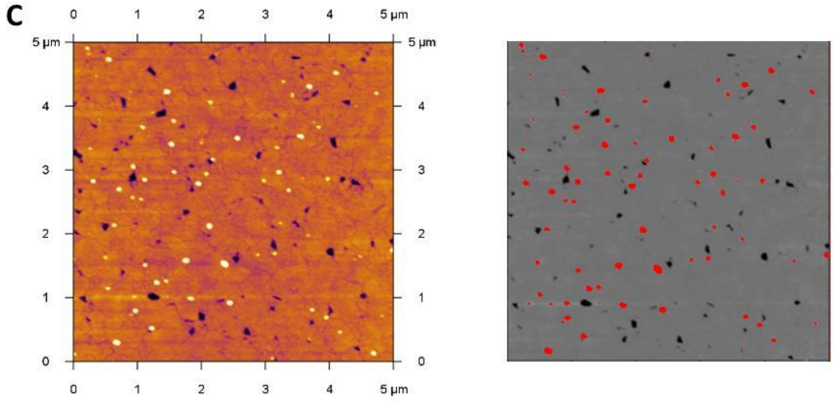


| Sample | Area Coverage |
|---|---|
| 100% Methylated Jurkat | 1.34% |

**Supplementary Figure 5.** 2D-AFM image of DNA with different methylation status. A) Unmethylated WGA DNA B) Hypomethylated BT474 breast cancer cell line DNA (43% global methylation) C) 100% Methylated Jurkat DNA. Right tables show the approximate %area coverage obtained from ImageJ analysis

**Optimisation of the operating parameters for electrochemistry experiments:** We first optimised the time to obtain maximum current difference for the adsorption of BT474 breast cancer cell derived DNA and fully unmethylated WGA DNA. As shown in supplementary figure 6A, adsorption of both the DNA increases with increasing time. However, 10 min provided the maximum differences between the relative DPV current for BT474 and WGA DNA. Similarly, we optimised the DNA concentration and found that 10ng/µl sample provided significant difference between the relative DPV current for the BT474 and WGA DNA (supplementary figure 6B).

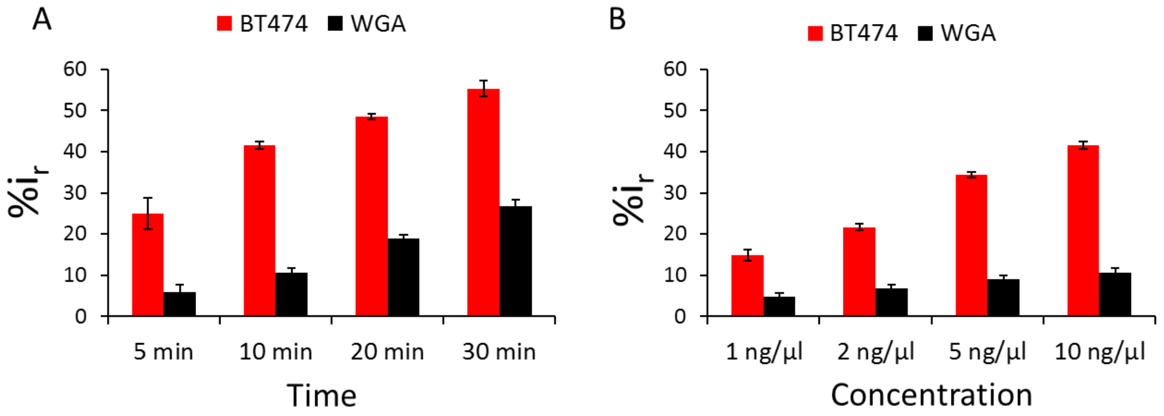


**Supplementary Figure 6.** Optimisation of the operating parameters for cell line DNA. Mean values of the relative DPV current obtained for the adsorption of WGA and BT474 cell derived DNA at (A) different time (DNA concentration, 10ng/µl) and (B) different concentration (adsorption time, 10 min). Each bar represents the average of three separate trials (n = 3). Error bars represent the standard deviation of measurements (relative standard deviation (%RSD) was found to be <5% for n = 3).

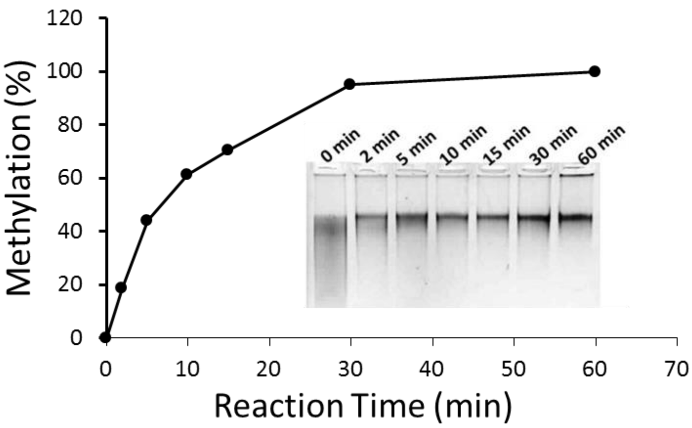


| Reaction Time (Min) | Methylation (%) |
|---|---|
| 0 | 0 |
| 2 | 18.85 |
| 5 | 43.99 |
| 10 | 61.36 |
| 15 | 70.59 |
| 30 | 95.09 |
| 60 | 100 |

**Supplementary Figure 7**. ImageJ analysis of the electrophoresis gel picture showing the methylation levels for each of the samples treated with *M.SssI* enzyme with different reaction time (methylation level is assumed 100% for 60 min reaction times as suggested by the manufacturer instruction for the kit used).

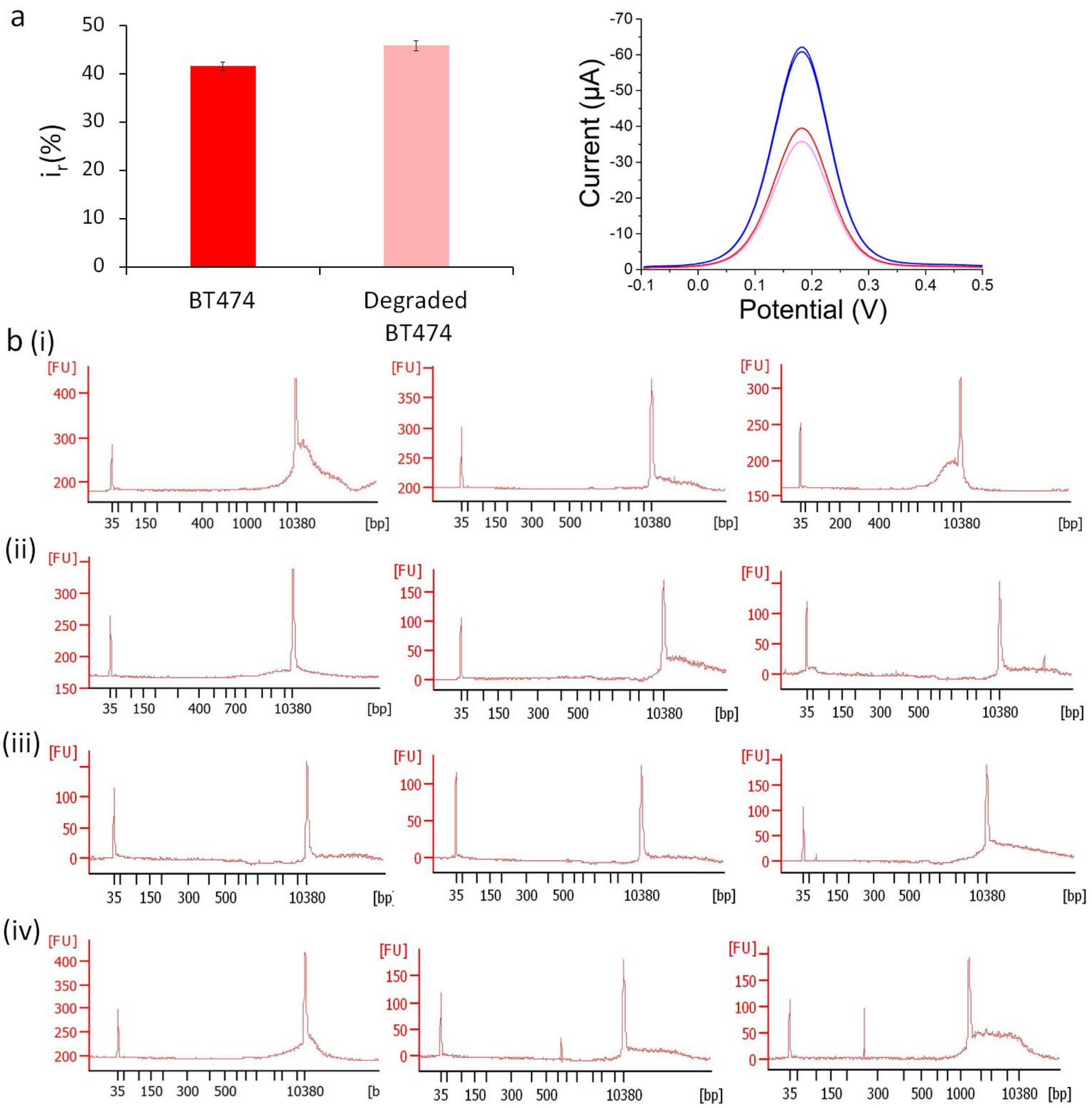


**Supplementary Figure 8. DNA fragmentation measurement and it's effect on adsorption** (a) Bars represent the relative current mean values (%$i_r$) for BT474 Breast cancer cell derived DNA (Red Bar) and fragmented BT474 DNA (Pink Bar). Each data point represents the average of three separate trials, and error bars represent the standard deviation of measurements (%RSD =<5% for n = 3). Right Panel: Corresponding DPV graphs and their respective baselines. (b)Bioanalyser sensogram showing the size (no. of base pairs) of the DNA. (i) Three breast cancer patient tissue DNA, Supplementary Table 6, Sample No. 1-3, (ii) Three lymphoma cancer patient tissue DNA, Supplementary Table 7, Sample No. 55-57,(iii) Three prostate cancer patient tissue DNA, Supplementary Table 8, Sample No. 65-67, iv) Three normal tissue DNA, Supplementary Table 9, Sample No. 1 (Normal Breast), 20 (Normal Lymphnode), 23 (Normal Prostate).

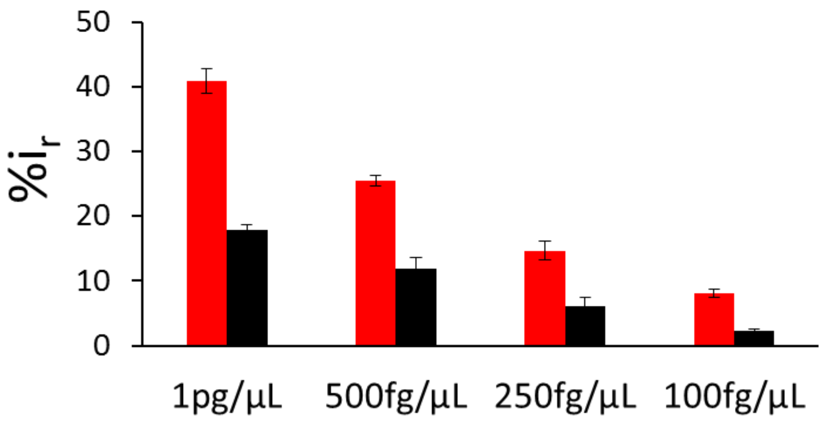


**Supplementary Figure 9.** Sensitivity for the detection of DNA methylation landscape in cfDNA. Mean values of the relative DPV current obtained for the adsorption of 5 µl of cfDNA samples derived from the blood plasma of patient (Supplementary Table 10, Sample number 2) and healthy (Supplementary Table 11, Sample number 7) individuals at different concentration. Each bar represents the average of three separate trials (n = 3). Error bars represent the standard deviation of measurements (relative standard deviation (%RSD) was found to be <5% for n = 3).

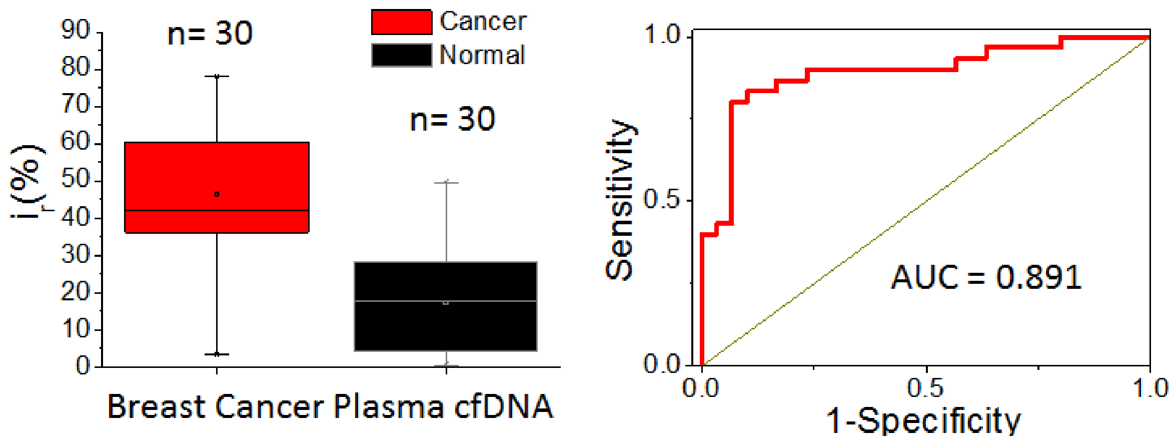


**Supplementary Figure 10.** Box plot showing the mean relative current values generated by electrochemical detection of plasma cfDNAs extracted from 30 normal and 30 breast cancer patients. The ROC analysis is shown on the right. In the box and whisker plots, the middle lines of the boxes represent the median (50th percentile) and the terminal line of the boxes represents the 25th to 75th percentile. The whiskers represent the lowest and the highest value.

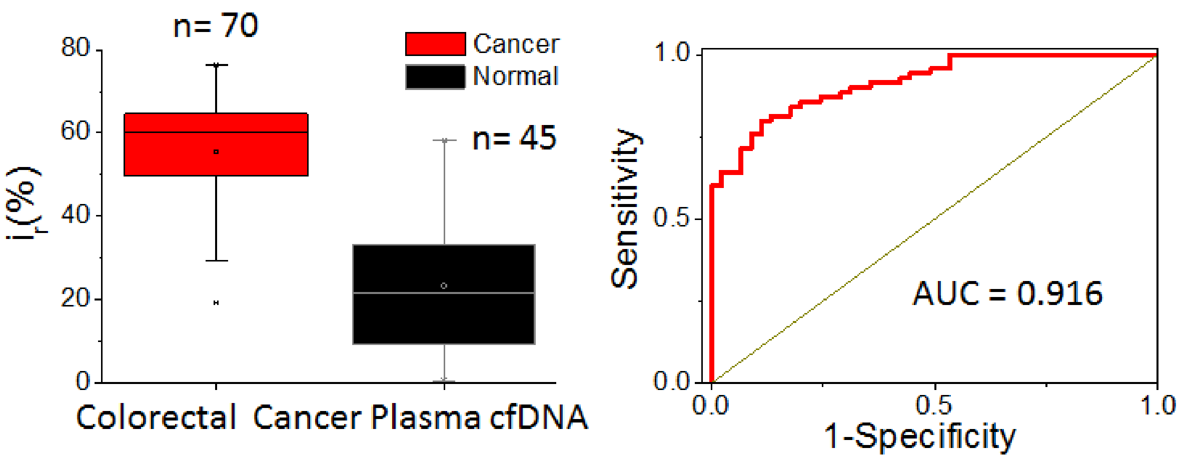


**Supplementary Figure 11.** Box plot showing the mean relative current values generated by electrochemical detection of plasma cfDNAs extracted from 45 normal and 70 colorectal cancer patients. The ROC analysis are shown on the right. In the box and whisker plots, the middle lines of the boxes represent the median (50th percentile) and the terminal line of the boxes represents the 25th to 75th percentile. The whiskers represent the lowest and the highest value.

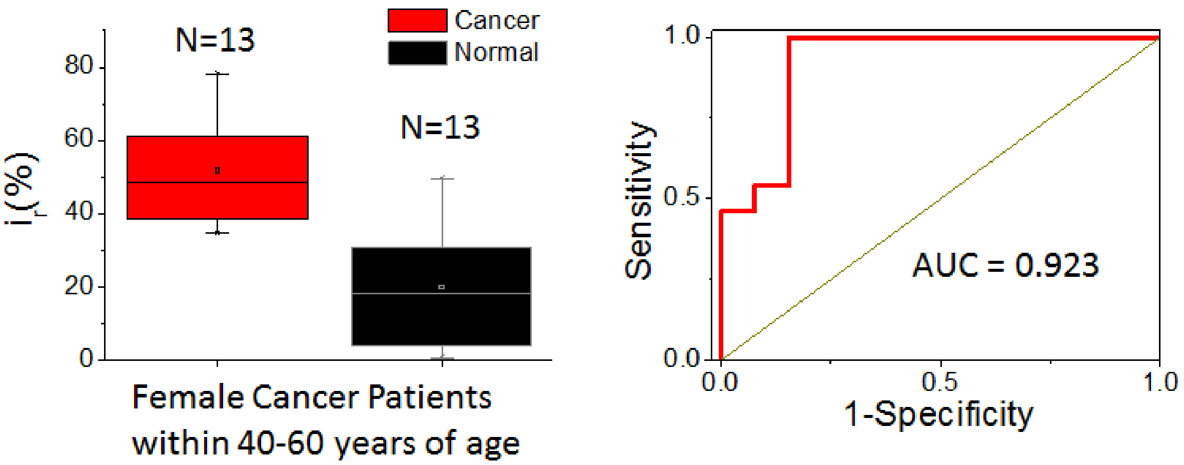


**Supplementary Figure 12.** Box plot showing the mean relative current values generated by electrochemical detection of plasma cfDNAs extracted from 13 normal and 13 female cancer patients with the age above 40 years. The ROC analysis is shown on the right. In the box and whisker plots, the middle lines of the boxes represent the median (50th percentile) and the terminal line of the boxes represents the 25th to 75th percentile. The whiskers represent the lowest and the highest value.

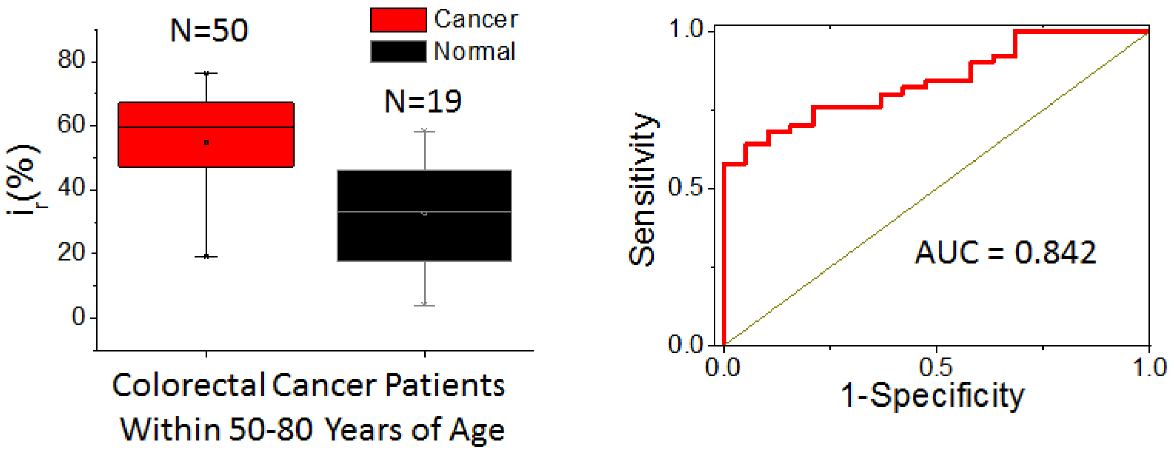


**Supplementary Figure 13.** Box plot showing the mean relative current values generated by electrochemical detection of plasma cfDNAs extracted from 19 healthy individuals and 50 colorectal cancer patients within 50-80 years of age. The ROC analysis is shown on the right. In the box and whisker plots, the middle lines of the boxes represent the median (50th percentile) and the terminal line of the boxes represents the 25th to 75th percentile. The whiskers represent the lowest and the highest value.

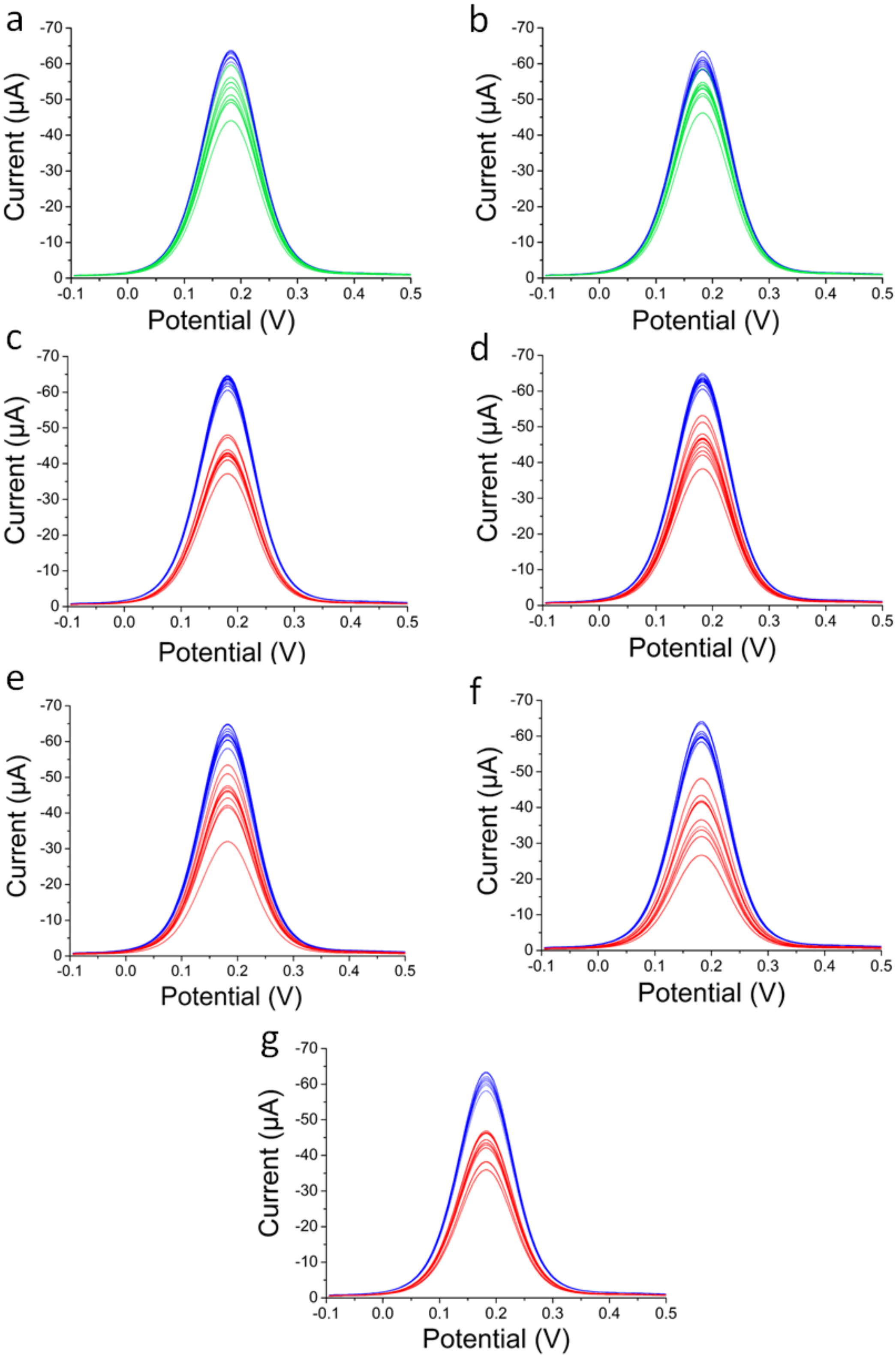


**Supplementary Figure 14:** DPV signals for the adsorption of gDNA derived from 31 normal breast tissues (green), 54 breast cancer tissues (red), and their corresponding baselines (blue). a) Normal Samples 1-15 b) Normal Samples 16-31 c) Patient Sample 1-11 d) Patient Samples 12-22 e) Patient Samples 22-33 f) Patient Samples 34-45 g) Patient sample 34-54.

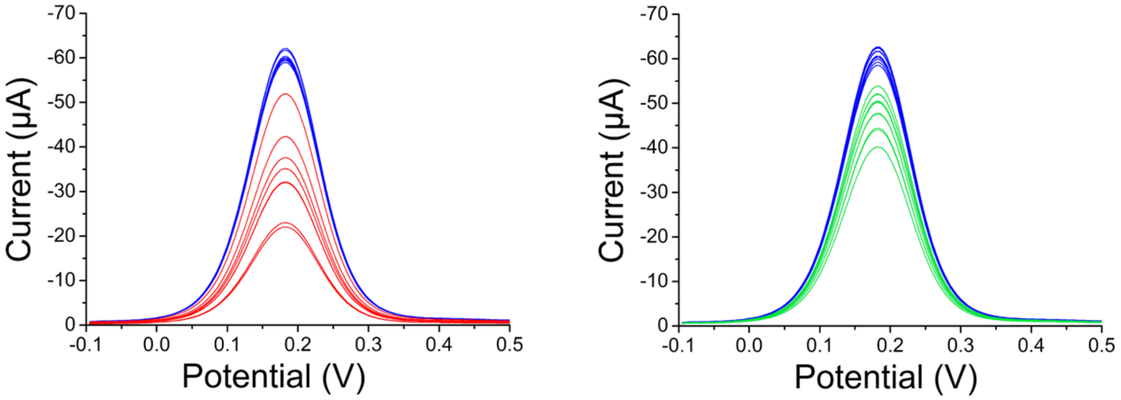


**Supplementary Figure 15:** DPV signals for the adsorption of gDNA derived from 8 prostate cancer tissues (red), 10 normal prostate tissues (green) and their corresponding baselines (blue).

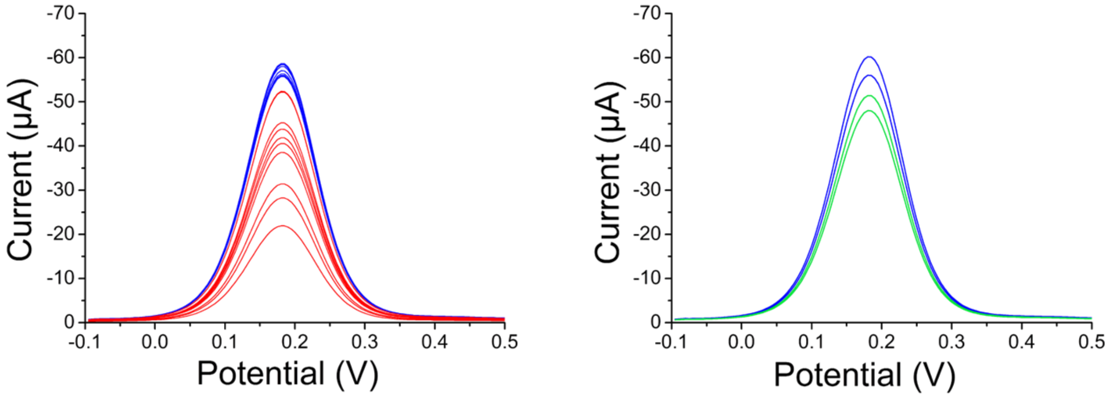


**Supplementary Figure 16:** DPV signals for the adsorption of gDNA derived from 10 lymphoma cancer tissues (red), 2 normal lymphoma tissues (green) and their corresponding baselines (blue).

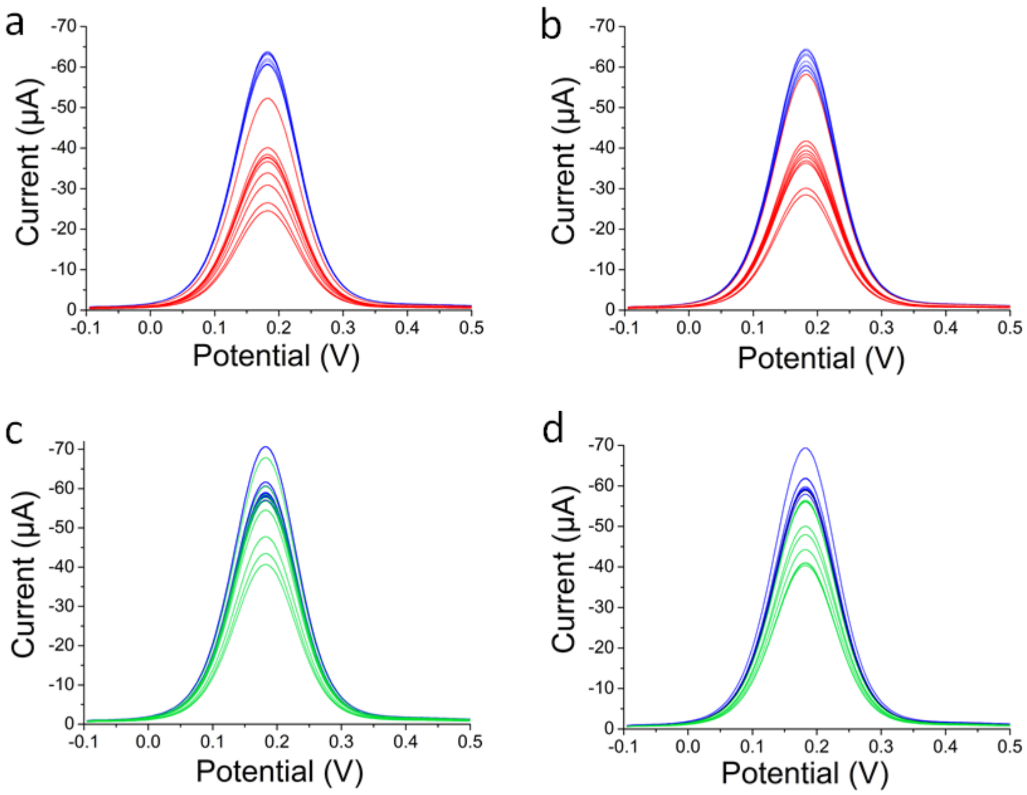


**Supplementary Figure 17:** DPV signals for the adsorption of cfDNA derived from 20 breast cancer plasma (red), 20 healthy plasma (green) and their corresponding baselines (blue).a) Patient samples 1-10 b) Patient samples 11-20 c) Normal samples 1-10 d) normal samples 11-20

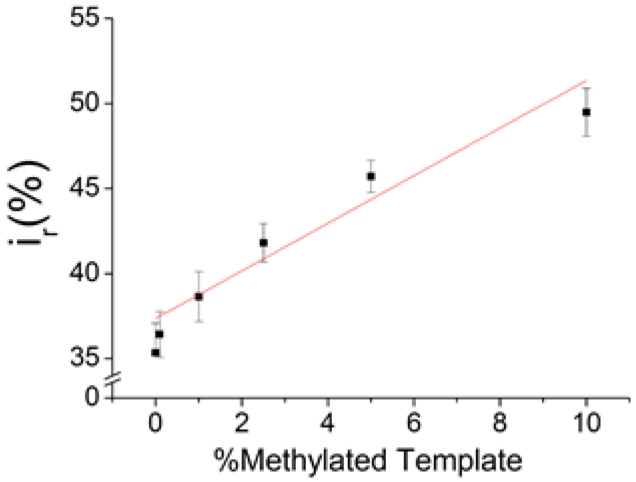


**Supplementary Figure 18:** relative adsorption (%$i_r$) vs % Methylated template DNA plot showing that the adsorption of normal cfDNA increases with the increased amount of clustered methylated DNA spiked in the normal cfDNA sample solution. Each point

represents the average of three separate trials (n = 3). Error bars represent the standard deviation of measurements (relative standard deviation (%RSD) was found to be <5% for n = 3).

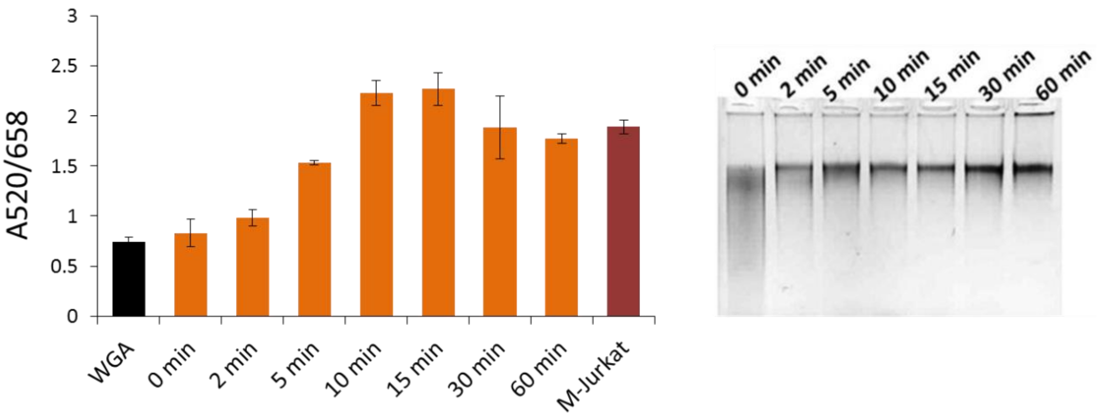


**Supplementary Figure 19:** Effect of non-clustered CpG methylation on gold-DNA adsorption. Relative absorbance ($A_{520/658}$) values for unmethylated (WGA), fully methylated (Jurkat) DNAs, and various genomic DNAs prepared from WGA DNA by enzymatic reaction using the M.SssI CpG methyltransferase enzyme for increasing time periods up to 60 min. Each bar represents the average of three separate trials (n = 3). Error bars represent the standard deviation of measurements (relative standard deviation (%RSD) was found to be <5% for n = 3). The inset panel shows an electrophoresis gel of the enzymatically methylated DNA samples digested with methylation sensitive HpaII restriction enzyme.

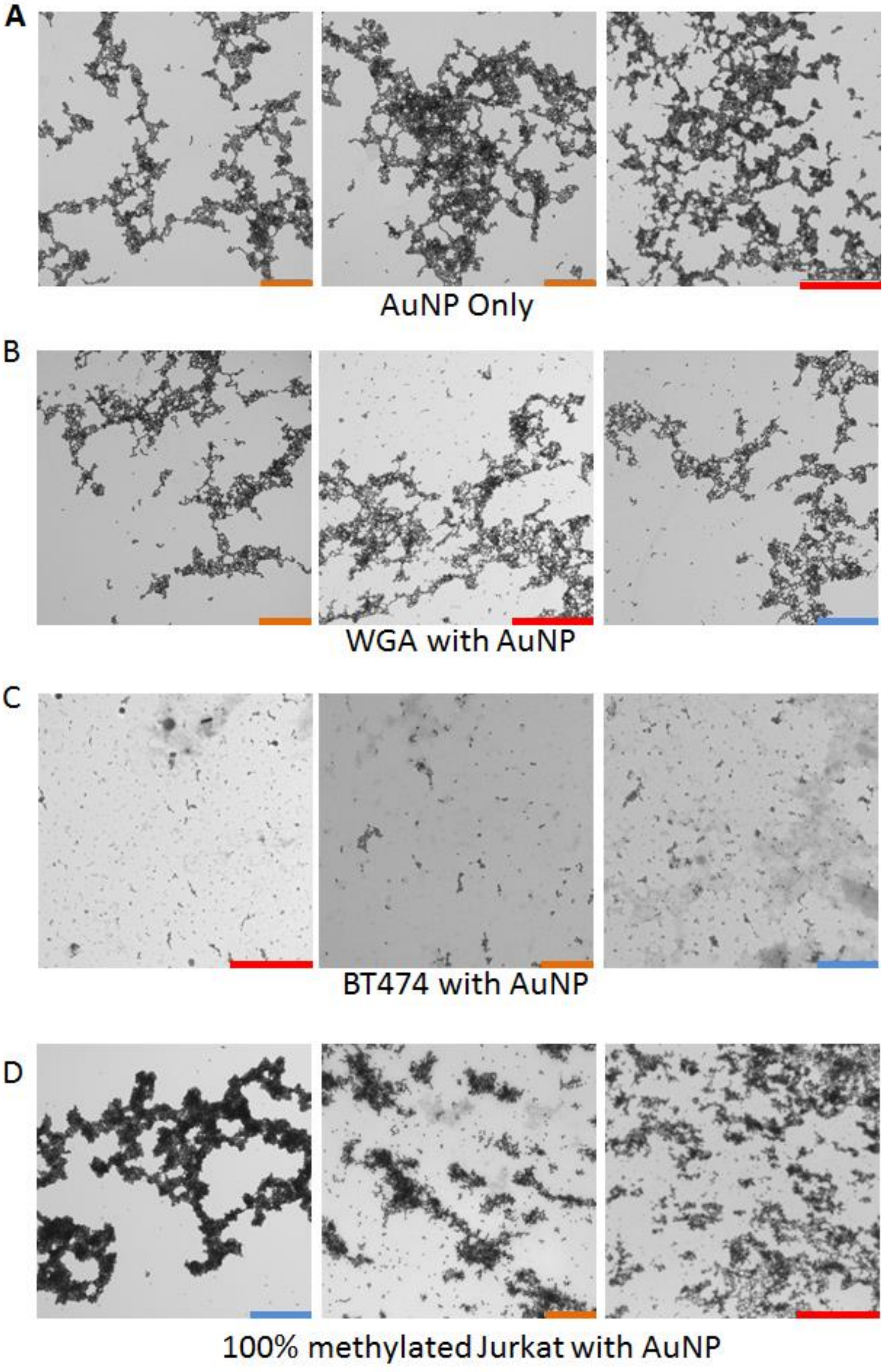


**Supplementary Figure 20**. TEM images for gold Nanoparticles and their interaction with DNA. Scale bars are 2000nm (red) 1000nm (blue) and 500nm (orange) for all the supplementary figures.

A

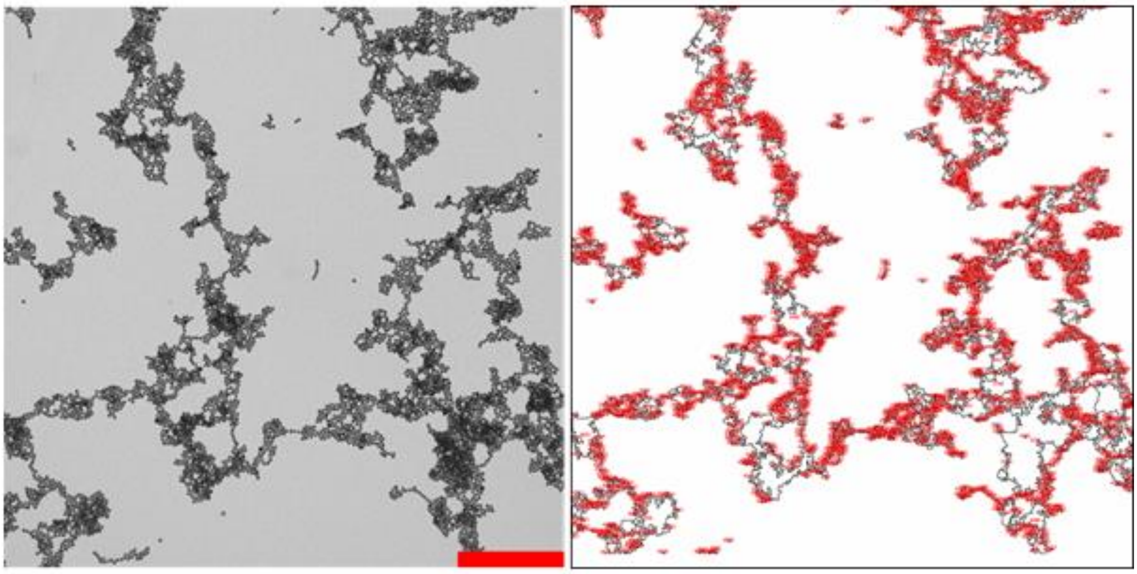

| Sample | Aggregates Count | Total Area ($nm^2$) | Average Size ($nm^2$) | %Area |
|---|---|---|---|---|
| AuNP | 2270 | 1067996.107 | 470.483 | 13.096 |

B

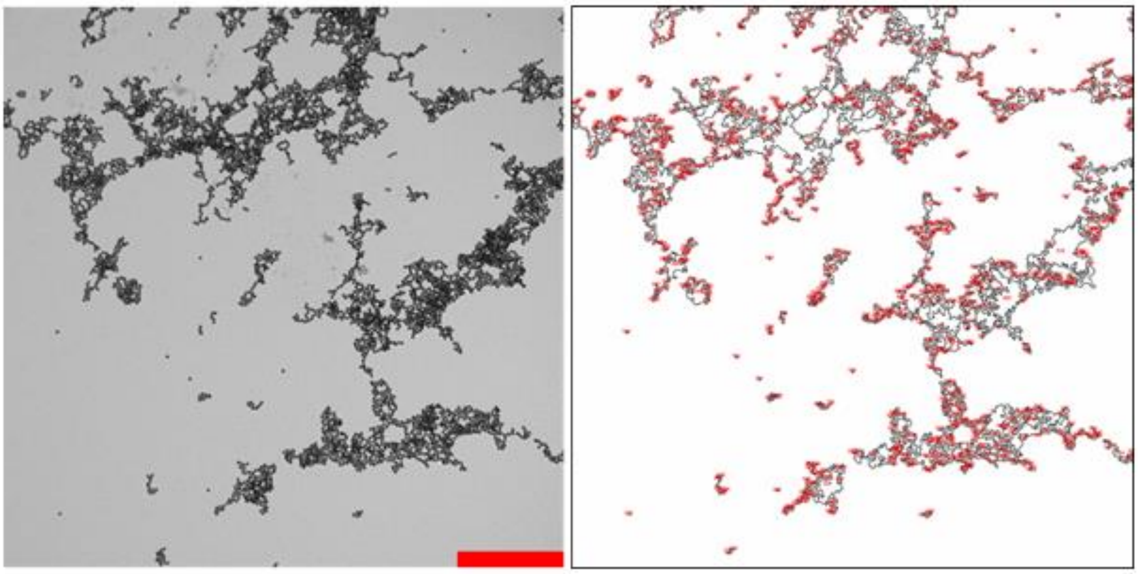

| Sample | Aggregates Count | Total Area ($nm^2$) | Average Size ($nm^2$) | %Area |
|---|---|---|---|---|
| WGA+ AuNP | 979 | 808571.931 | 825.916 | 9.915 |

C

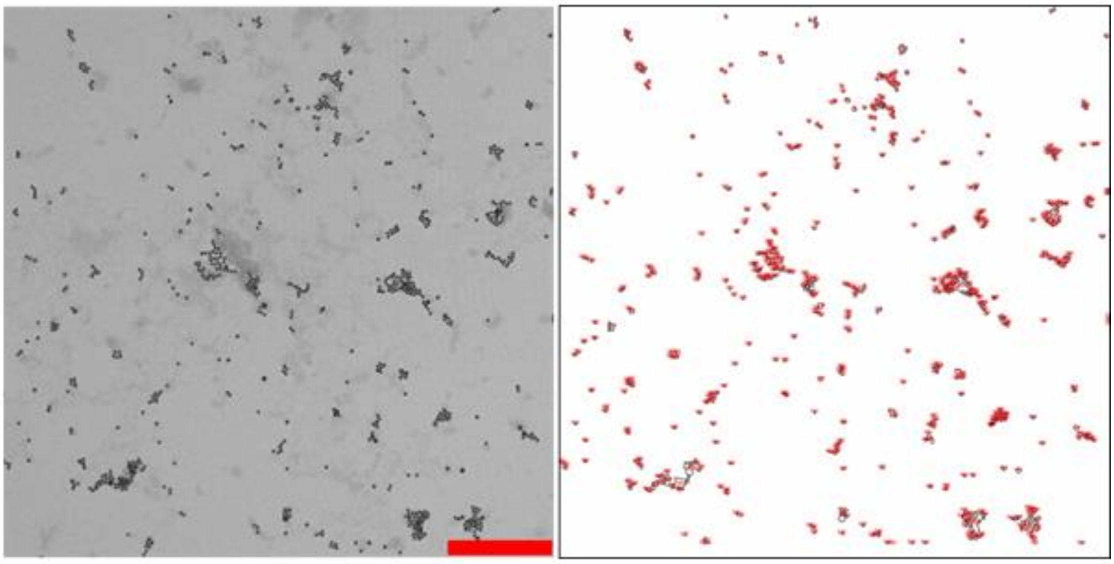

| Sample | Aggregates Count | Total Area ($nm^2$) | Average Size ($nm^2$) | %Area |
|---|---|---|---|---|
| BT474+ AuNP | 559 | 120000 | 214.47 | 1.47 |

D

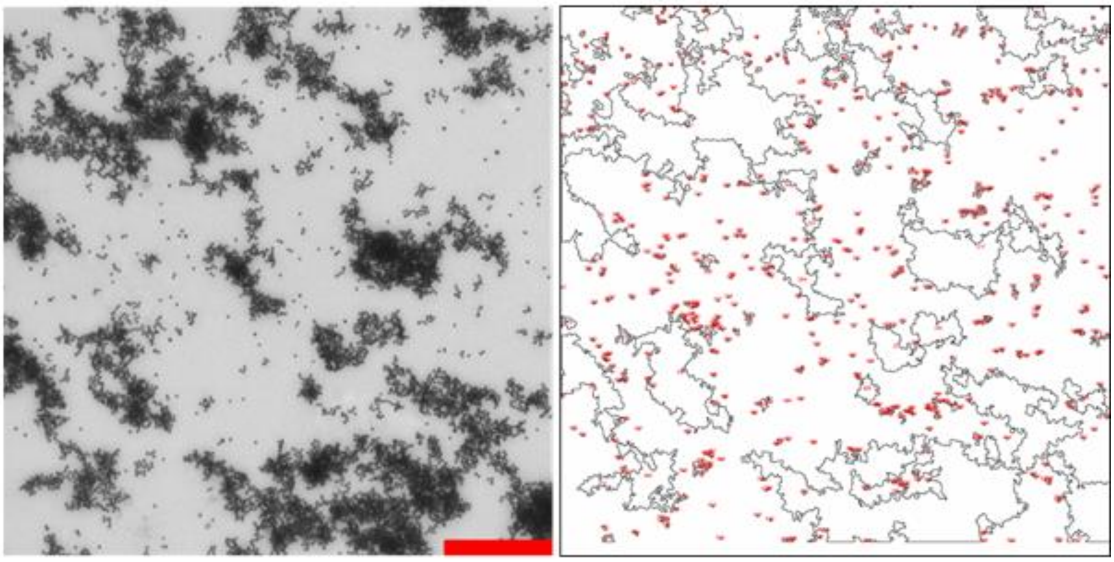

| Sample | Aggregates Count | Total Area ($nm^2$) | Average Size ($nm^2$) | %Area |
|---|---|---|---|---|
| 100% Meth Jurkat+ AuNP | 557 | 2110000 | 3791.893 | 25.999 |

**Supplementary Figure 21**. ImageJ analysis for TEM image for AuNP and its interaction with gDNA having different methylation status. (A) AuNP (B) AUNP with WGA DNA (C)AuNP with BT474 DNA (D) AuNP with Jurkat 100% methylated DNA. Scale bars are 500nm for all the supplementary figures.

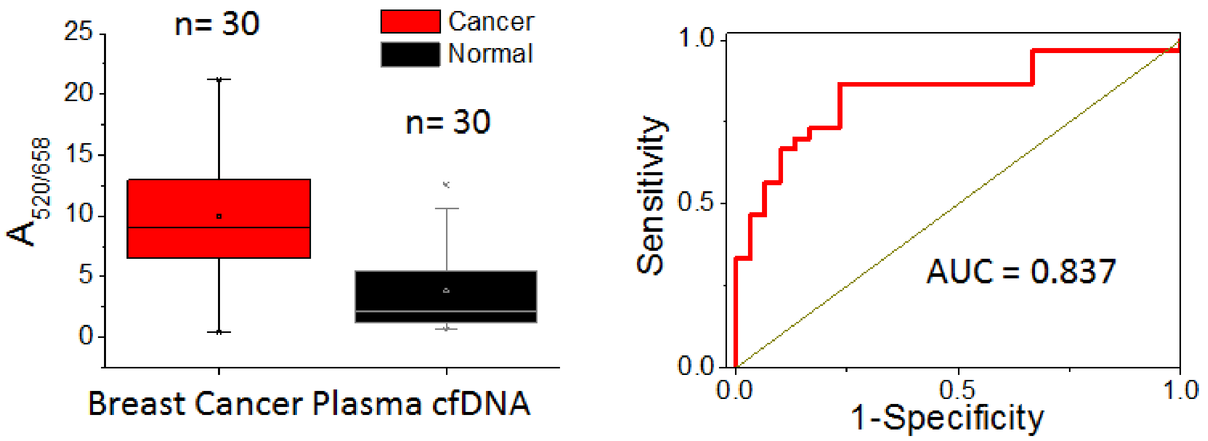


**Supplementary Figure 22.** Box plot showing the mean relative absorbance values $A_{520/658}$ of AuNP-cfDNA solution for cfDNA samples derived from the plasma of breast cancer patients or healthy donors. The ROC analysis is shown on the right. In the box and whisker plots, the middle lines of the boxes represent the median (50th percentile) and the terminal line of the boxes represents the 25th to 75th percentile. The whiskers represent the lowest and the highest value.

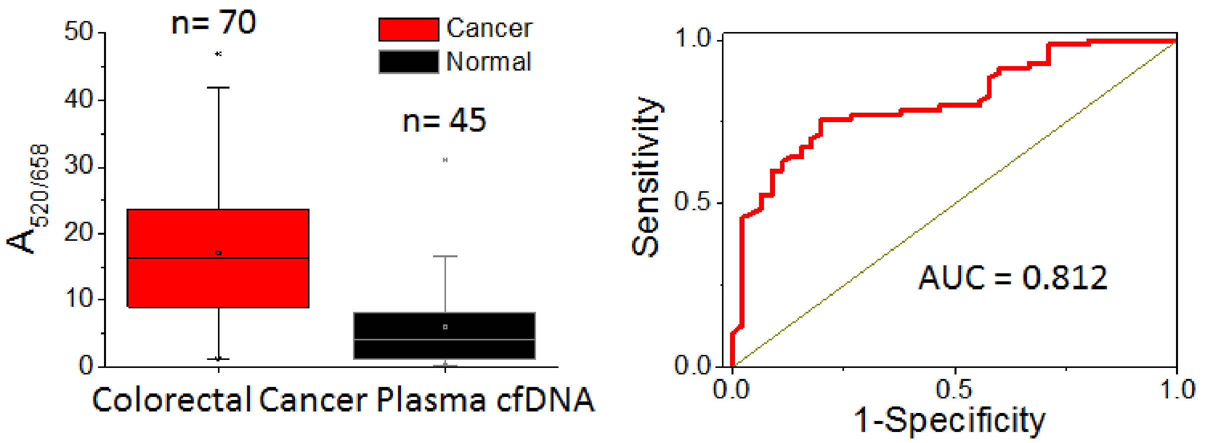


**Supplementary Figure 23.** Box plot showing the mean relative absorbance values $A_{520/658}$ of AuNP-cfDNA solution for cfDNA samples derived from the plasma of colorectal cancer patients or healthy donors. The ROC analysis is shown on the right. In the box and whisker plots, the middle lines of the boxes represent the median (50th percentile) and the terminal line of the boxes represents the 25th to 75th percentile. The whiskers represent the lowest and the highest value.

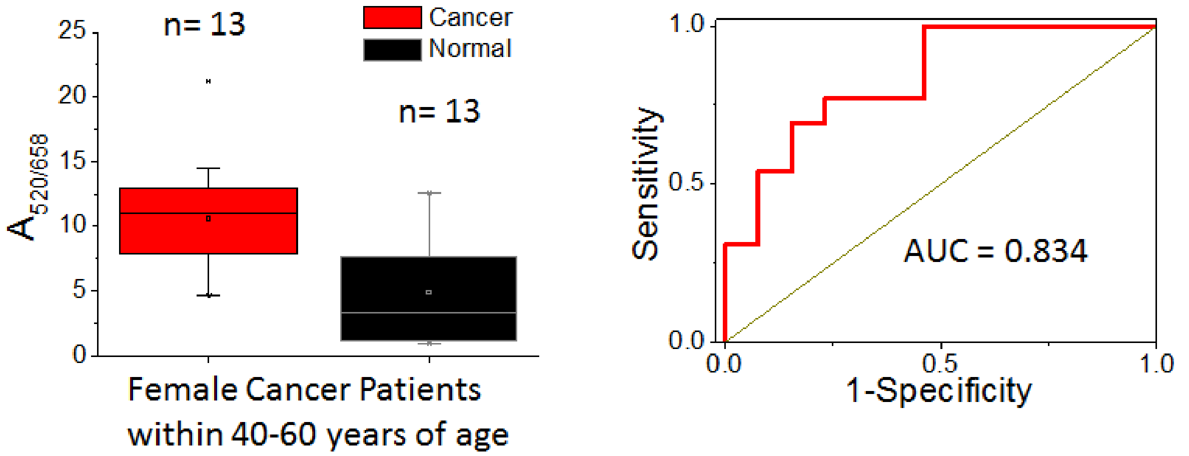


**Supplementary Figure 24.** Box plot showing the mean relative absorbance values $A_{520/658}$ of AuNP-cfDNA solution for cfDNA samples derived from the plasma of female cancer patients or healthy donors within 40-60 years of age. The ROC analysis is shown on the right. In the box and whisker plots, the middle lines of the boxes represent the median (50th percentile) and the terminal line of the boxes represents the 25th to 75th percentile. The whiskers represent the lowest and the highest value.

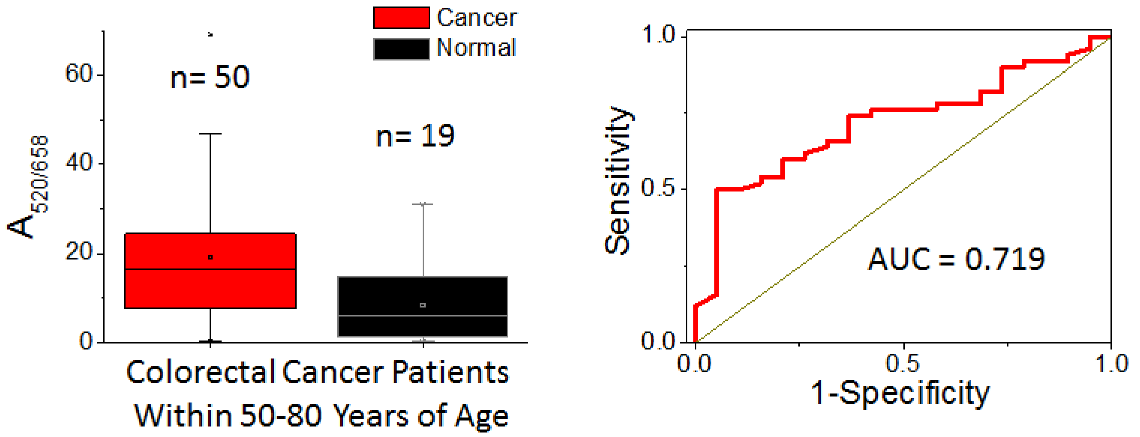


**Supplementary Figure 25.** Box plot showing the mean relative absorbance values $A_{520/658}$ of AuNP-cfDNA solution for cfDNA samples derived from the plasma of colorectal cancer patients or healthy donors within 50-80 years of age. The ROC analysis is shown on the right. In the box and whisker plots, the middle lines of the boxes represent the median (50th percentile) and the terminal line of the boxes represents the 25th to 75th percentile. The whiskers represent the lowest and the highest value.

## Supplementary Tables

**Supplementary Table 1. P value obtained from the student's t- test for the analysis of paired normal and cancer DNA derived from tissue and plasma samples**

| **Sample Tissue DNA-Electrochemistry (Methylscape)** | **P value** |
|---|---|
| All Cancer tissue DNA vs All Normal tissue DNA | 0.0015 |
| Breast Cancer tissue DNA vs normal tissue DNA | 0.0001 |
| Lymphoma tissue DNA vs normal tissue DNA | 0.2547 |
| Prostate cancer tissue DNA vs normal tissue DNA | 0.0012 |
| **Sample Plasma DNA-Electrochemistry** | |
| Breast and Colorectal Cancer Plasma DNA vs Normal Plasma DNA | 0.0001 |
| Sample **Tissue and Plasma DNA- Nanoparticle (Nanomethylscape)** | |
| All Cancer tissue DNA vs Normal tissue DNA | 0.0005 |
| Breast and Colorectal Cancer Plasma DNA vs Normal Plasma DNA | 0.0001 |

**Supplementary Table 2. Statistical Diagnostic Test evaluation for electrochemical analysis of tissue derived gDNA. (Cut off %$i_r$ = 20, Cancer>20<normal)**

| **Statistic** | **Value for electrochemical gDNA test** |
|---|---|
| Sensitivity | 93.06% |
| Specificity | 80.65% |
| Disease Prevalence (DP) | 69.90% |
| Positive Predictive Value (PPV) | 91.78% |
| Negative Predictive Value(NPV) | 83.33% |
| Accuracy | 89.32% |

**Supplementary Table 3. Statistical Diagnostic Test evaluation for electrochemical analysis of plasma derived cfDNA. (Cut off %$i_r$ = 35.7, Cancer>35.7<normal)**

| Statistic | Value for electrochemical cfDNA test |
|---|---|
| Sensitivity | 84.00% |
| Specificity | 82.22% |
| Disease Prevalence (DP) | 68.97% |
| Positive Predictive Value (PPV) | 91.30% |
| Negative Predictive Value(NPV) | 69.81% |
| Accuracy | 83.45% |

**Supplementary Table 4. Statistical Diagnostic Test evaluation for nanoparticle based analysis of of tissue derived gDNA. (Cut off %$i_r$ = 35.7, Cancer>35.7<normal)**

| Statistic | Value for colorimetric gDNA test |
|---|---|
| Sensitivity | 70.83% |
| Specificity | 83.33% |
| Disease Prevalence (DP) | 50.00% |
| Positive Predictive Value (PPV) | 80.95% |
| Negative Predictive Value(NPV) | 74.07% |
| Accuracy | 77.08% |

**Supplementary Table 5. Statistical Diagnostic Test evaluation for nanoparticle based analysis of plasma derived cfDNA. (Cut off $A_{520/658}$ = 8.7, Cancer>8.7<normal)**

| Statistic | Value for colorimetric cfDNA test |
|---|---|
| Sensitivity | 70.00% |
| Specificity | 80.00% |
| Disease Prevalence (DP) | 68.97% |
| Positive Predictive Value (PPV) | 88.61% |
| Negative Predictive Value(NPV) | 54.55% |
| Accuracy | 73.10% |

**Supplementary Table 6. Clinical Information of Breast Cancer Patients (for tissue derived gDNA samples) *N/A= Not Available**

| Sample Number | Gender | Age (Yrs) | Cancer Type | Cancer Stage | Global Methylation (%) | Mean Relative Adsorption (%$i_r$) |
|---|---|---|---|---|---|---|
| 1 | Female | 56 | ER+ Breast | Metastatic | 33.21 | 30.98 |
| 2 | Female | 76 | ER+ Breast | Metastatic | 41.46 | 39.69 |
| 3 | Female | 65 | ER+ Breast | Metastatic | 35.64 | 34.25 |
| 4 | Female | 71 | ER+ Breast | Metastatic | 39.25 | 33.66 |
| 5 | Female | 53 | ER+ Breast | Metastatic | 48.70 | 26.64 |
| 6 | Female | 44 | ER+ Breast | Metastatic | 45.32 | 32.02 |
| 7 | Female | N/A | ER+ Breast | Metastatic | 47.61 | 28.96 |
| 8 | Female | 63 | ER+ Breast | Metastatic | 34.28 | 32.69 |
| 9 | Female | 63 | ER+ Breast | Metastatic | 35.26 | 25.95 |
| 10 | Female | 59 | ER+ Breast | Metastatic | 46.14 | 35.97 |
| 11 | Female | 55 | ER+ Breast | Metastatic | 57.70 | 27.90 |
| 12 | Female | 85 | ER+ Breast | Metastatic | 48.93 | 32.03 |
| 13 | Female | 69 | ER+ Breast | Metastatic | 43.52 | 15.28 |
| 14 | Female | 66 | ER+ Breast | Metastatic | 34.61 | 25.95 |
| 15 | Female | 60 | ER+ Breast | Metastatic | Error reading | 32.11 |
| 16 | Female | 49 | ER+ Breast | Metastatic | 54.61 | 19.29 |
| 17 | Female | 66 | ER+ Breast | Metastatic | 43.68 | 27.42 |
| 18 | Female | 84 | ER+ Breast | Metastatic | 33.50 | 37.83 |
| 19 | Female | 60 | ER+ Breast | Metastatic | 39.20 | 26.10 |
| 20 | Female | 71 | ER+ Breast | Metastatic | 51.64 | 29.96 |
| 21 | Female | 64 | ER+ Breast | Metastatic | 53.61 | 22.81 |
| 22 | Female | 76 | ER+ Breast | Metastatic | 31.48 | 33.37 |
| 23 | Female | 66 | ER+ Breast | Metastatic | 56.82 | 22.11 |
| 24 | Female | 58 | ER+ Breast | Metastatic | 49.62 | 18.23 |
| 25 | Female | 64 | ER+ Breast | Metastatic | 36.59 | 30.42 |
| 26 | Female | 60 | ER+ Breast | Metastatic | 37.43 | 27.51 |
| 27 | Female | 46 | ER+ Breast | Metastatic | 42.90 | 25.92 |
| 28 | Female | 73 | ER+ Breast | Metastatic | 45.27 | 24.52 |
| 29 | Female | 52 | ER+ Breast | Metastatic | 40.20 | 23.62 |
| 30 | Female | 66 | ER+ Breast | Metastatic | No Sample left | 43.05 |
| 31 | Female | 53 | ER+ Breast | Metastatic | 37.63 | 31.28 |
| 32 | Female | 63 | ER+ Breast | Metastatic | 46.29 | 26.22 |
| 33 | Female | 66 | ER+ Breast | Metastatic | No Sample left | 30.70 |
| 34 | Female | 55 | ER+ Breast | Metastatic | 34.57 | 44.91 |
| 35 | Female | 57 | ER+ Breast | Metastatic | 62.80 | 25.66 |
| 36 | Female | N/A | ER+ Breast | Metastatic | No Sample left | 39.66 |
| 37 | Female | N/A | ER+ Breast | Metastatic | No Sample left | 32.38 |

| | | | | | | |
|---|---|---|---|---|---|---|
| 38 | Female | N/A | ER+ Breast | Metastatic | No Sample left | 57.44 |
| 39 | Female | N/A | ER+ Breast | Metastatic | No Sample left | 34.05 |
| 40 | Female | N/A | ER+ Breast | Metastatic | No Sample left | 44.28 |
| 41 | Female | N/A | ER+ Breast | Metastatic | No Sample left | 47.56 |
| 42 | Female | N/A | ER+ Breast | Metastatic | No Sample left | 21.84 |
| 43 | Female | N/A | ER+ Breast | Metastatic | No Sample left | 24.21 |
| 44 | Female | N/A | ER+ Breast | Metastatic | 28.23 | 32.02 |
| 45 | Female | N/A | ER+ Breast | Metastatic | No Sample left | 28.96 |
| 46 | Female | N/A | ER+ Breast | Metastatic | No Sample left | 26.48 |
| 47 | Female | N/A | ER+ Breast | Metastatic | No Sample left | 26.51 |
| 48 | Female | N/A | ER+ Breast | Metastatic | No Sample left | 40.34 |
| 49 | Female | N/A | ER+ Breast | Metastatic | No Sample left | 21.50 |
| 50 | Female | N/A | ER+ Breast | Metastatic | 27.33 | 37.59 |
| 51 | Female | N/A | ER+ Breast | Metastatic | No Sample left | 32.16 |
| 52 | Female | N/A | ER+ Breast | Metastatic | No Sample left | 25.73 |
| 53 | Female | N/A | ER+ Breast | Metastatic | No Sample left | 40.63 |
| 54 | Female | N/A | ER+ Breast | Metastatic | No Sample left | 30.28 |

**Supplementary Table 7. Clinical Information of Lymphoma Patients (for tissue derived gDNA samples)**

| | | | | | | |
|---|---|---|---|---|---|---|
| 55 | Female | 78 | Lymphoma | Metastatic | 45.77 | 9.57 |
| 56 | Female | 65 | Lymphoma | Metastatic | 43.89 | 30.25 |
| 57 | Female | 75 | Lymphoma | Metastatic | 47.22 | 8.58 |
| 58 | Male | 72 | Lymphoma | Metastatic | 59.40 | 27.92 |
| 59 | Female | 71 | Lymphoma | Metastatic | 45.63 | 62.43 |
| 60 | Female | 81 | Lymphoma | Metastatic | 33.60 | 33.51 |
| 61 | Female | 40 | Lymphoma | Metastatic | 30.48 | 45.14 |
| 62 | Female | 73 | Lymphoma | Metastatic | 40.26 | 50.56 |
| 63 | Male | 49 | Lymphoma | Metastatic | 42.67 | 24.18 |
| 64 | Female | 73 | Lymphoma | Metastatic | 37.24 | 22.73 |

**Supplementary Table 8. Clinical Information of Prostate Cancer Patients (for tissue derived gDNA samples)**

| | | | | | | |
|---|---|---|---|---|---|---|
| 65 | Male | 60 | Prostate | Metastatic | 31.20 | 62.80 |
| 66 | Male | 67 | Prostate | Metastatic | 37.63 | 31.65 |
| 67 | Male | 58 | Prostate | Metastatic | 42.54 | 12.86 |
| 68 | Male | 77 | Prostate | Metastatic | 41.87 | 37.16 |
| 69 | Male | 60 | Prostate | Metastatic | 56.21 | 41.95 |
| 70 | Male | 79 | Prostate | Metastatic | 32.83 | 46.42 |
| 71 | Male | 68 | Prostate | Metastatic | 34.59 | 61.39 |
| 72 | Male | 65 | Prostate | Metastatic | 37.68 | 48.51 |

**Supplementary Table 9. Clinical Information of Healthy Individuals (for tissue derived gDNA samples)**

| Sample Number | Gender | Age | Tissue Type | Global Methylation (%) | Mean Relative Adsorption (%$i_r$) |
|---|---|---|---|---|---|
| 1 | Female | 38 | Normal Breast | 74.29 | 17.96 |
| 2 | Female | 22 | Normal Breast | No Sample left | 12.66 |
| 3 | Female | 38 | Normal Breast | No Sample left | 5.47 |
| 4 | Female | 53 | Normal Breast | 63.51 | 16.60 |
| 5 | Female | 58 | Normal Breast | No Sample left | 10.09 |
| 6 | Female | 46 | Normal Breast | No Sample left | 11.14 |
| 7 | Female | 38 | Normal Breast | No Sample left | 17.68 |
| 8 | Female | 21 | Normal Breast | 56.74 | 27.50 |
| 9 | Female | 50 | Normal Breast | No Sample left | 19.38 |
| 10 | Female | 53 | Normal Breast | 60.91 | 17.15 |
| 11 | Female | 56 | Normal Breast | No Sample left | 16.70 |
| 12 | Female | 34 | Normal Breast | No Sample left | 21.99 |
| 13 | Female | N/A | Normal Breast | No Sample left | 11.65 |
| 14 | Female | N/A | Normal Breast | No Sample left | 7.98 |
| 15 | Female | N/A | Normal Breast | No Sample left | 10.71 |
| 16 | Female | N/A | Normal Breast | No Sample left | 11.28 |
| 17 | Female | N/A | Normal Breast | No Sample left | 7.87 |
| 18 | Female | N/A | Normal Breast | No Sample left | 10.89 |
| 19 | Female | N/A | Normal Breast | 60.59 | 10.95 |
| 20 | Female | 30 | Normal lymph node | 58.21 | 17.32 |
| 21 | Male | 56 | Normal lymph node | 61.24 | 15.10 |
| 22 | Male | 82 | Normal Prostate | 68.20 | 16.80 |
| 23 | Male | 72 | Normal Prostate | 51.43 | 35.72 |
| 24 | Male | 79 | Normal Prostate | 62.37 | 16.70 |
| 25 | Male | 44 | Normal Prostate | 55.81 | 26.48 |
| 26 | Male | 69 | Normal Prostate | 51.29 | 19.60 |
| 27 | Male | 91 | Normal Prostate | 49.23 | 17.08 |
| 28 | Male | 72 | Normal Prostate | 71.48 | 24.14 |
| 29 | Male | 59 | Normal Prostate | 42.65 | 13.68 |
| 30 | Male | 62 | Normal Prostate | 48.28 | 22.61 |
| 31 | Male | 76 | Normal Prostate | 57.60 | 15.43 |

**Supplementary Table 10. Clinical information of Breast cancer patient samples ( for cfDNA samples extracted from the plasma )**

| Sample Number | Gender | Age | Cancer Type | Cancer Stage | Mean Relative Adsorption (%$i_r$) | Mean Relative Absorbance ($A_{520/658}$) |
|---|---|---|---|---|---|---|
| 1 | Female | 85 | ER+ Breast | Metastatic | 42.43 | 6.75 |
| 2 | Female | 76 | ER+ Breast | Metastatic | 41.67 | 5.85 |
| 3 | Female | 44 | ER+ Breast | Metastatic | 56.78 | 7.88 |
| 4 | Female | 66 | ER+ Breast | Metastatic | 16.23 | 1.27 |
| 5 | Female | 66 | ER+ Breast | Metastatic | 39.18 | 6.56 |
| 6 | Female | 58 | ER+ Breast | Metastatic | 38.56 | 11.50 |
| 7 | Female | 64 | ER+ Breast | Metastatic | 36.17 | 1.19 |
| 8 | Female | 59 | ER+ Breast | Metastatic | 35.77 | 4.61 |
| 9 | Female | 85 | ER+ Breast | Metastatic | 51.14 | 6.00 |
| 10 | Female | 76 | ER+ Breast | Metastatic | 60.49 | 5.63 |
| 11 | Female | 57 | ER+ Breast | Metastatic | 39.01 | 4.88 |
| 12 | Female | 66 | ER+ Breast | Metastatic | 8.69 | 1.13 |
| 13 | Female | 66 | ER+ Breast | Metastatic | 52.50 | 6.00 |
| 14 | Female | 58 | ER+ Breast | Metastatic | 42.16 | 4.88 |
| 15 | Female | 64 | ER+ Breast | Metastatic | 42.30 | 5.75 |
| 16 | Female | 59 | ER+ Breast | Metastatic | 48.60 | 10.05 |
| 17 | Female | 76 | ER+ Breast | Metastatic | 29.31 | 8.46 |
| 18 | Female | 60 | ER+ Breast | Metastatic | 34.67 | 11.00 |
| 19 | Female | 60 | ER+ Breast | Metastatic | 36.52 | 9.17 |
| 20 | Female | 85 | ER+ Breast | Metastatic | 30.58 | 9.75 |
| 21 | Female | 49 | ER+ Breast | Metastatic | 73.64 | 14.5 |
| 22 | Female | 63 | ER+ Breast | Metastatic | 37.94 | 10.83 |
| 23 | Female | 71 | ER+ Breast | Metastatic | 3.37 | 0.38 |
| 24 | Female | 71 | ER+ Breast | Metastatic | 77.14 | 15.83 |
| 25 | Female | 60 | ER+ Breast | Metastatic | 57.58 | 12.00 |
| 26 | Female | 64 | ER+ Breast | Metastatic | 74.93 | 6.31 |
| 27 | Female | 58 | ER+ Breast | Metastatic | 78.26 | 13.00 |
| 28 | Female | 60 | ER+ Breast | Metastatic | 74.32 | 20.00 |
| 29 | Female | 46 | ER+ Breast | Metastatic | 61.13 | 13.00 |
| 30 | Female | 46 | ER+ Breast | Metastatic | 70.96 | 21.25 |

**Supplementary Table 11. Clinical information of Colorectal cancer patient samples ( for cfDNA samples extracted from the plasma )**

| Sample Number | Gender | Age | Cancer Type | Cancer Stage | Mean Relative Adsortion (%$i_r$) | Mean Relative Absorbance ($A_{520/658}$) |
|---|---|---|---|---|---|---|
| 1 | F | 23* | Colorectal | Metastatic | 49.71 | 14.5 |
| 2 | M | 43 | Colorectal | Metastatic | 63.06 | 23.7 |
| 3 | M | 49 | Colorectal | Metastatic | 50.91 | 16.3 |
| 4 | M | 51 | Colorectal | Metastatic | 63.85 | 60 |
| 5 | M | 54 | Colorectal | Metastatic | 52.65 | 15 |
| 6 | F | 56 | Colorectal | Metastatic | 72.30 | 22.3 |
| 7 | M | 56 | Colorectal | Metastatic | 61.33 | 21 |
| 8 | F | 56 | Colorectal | Metastatic | 68.05 | 47 |
| 9 | F | 56 | Colorectal | Metastatic | 61.10 | 66 |
| 10 | M | 59 | Colorectal | Metastatic | 63.30 | 0.4 |
| 11 | M | 61 | Colorectal | Metastatic | 50.40 | 10.8 |
| 12 | M | 61 | Colorectal | Metastatic | 29.23 | 14.8 |
| 13 | M | 61 | Colorectal | Metastatic | 67.08 | 36 |
| 14 | F | 62 | Colorectal | Metastatic | 61.96 | 28.5 |
| 15 | F | 62 | Colorectal | Metastatic | 62.70 | 16.3 |
| 16 | F | 63 | Colorectal | Metastatic | 62.00 | 10.6 |
| 17 | F | 63 | Colorectal | Metastatic | 73.43 | 22 |
| 18 | M | 63 | Colorectal | Metastatic | 69.64 | 15.7 |
| 19 | M | 64 | Colorectal | Metastatic | 70.14 | 21.7 |
| 20 | M | 64 | Colorectal | Metastatic | 63.70 | 67 |
| 21 | M | 65 | Colorectal | Metastatic | 58.03 | 9 |
| 22 | M | 65 | Colorectal | Metastatic | 54.45 | 31 |
| 23 | F | 65 | Colorectal | Metastatic | 47.19 | 0.7 |
| 24 | M | 65 | Colorectal | Metastatic | 20.40 | 2 |
| 25 | F | 66 | Colorectal | Metastatic | 35.85 | 0.9 |
| 26 | M | 69 | Colorectal | Metastatic | 25.70 | 2.1 |
| 27 | M | 69 | Colorectal | Metastatic | 26.80 | 1.8 |
| 28 | F | 70 | Colorectal | Metastatic | 76.16 | 3 |
| 29 | F | 70 | Colorectal | Metastatic | 35.96 | 69 |
| 30 | F | 70 | Colorectal | Metastatic | 59.55 | 15.3 |
| 31 | M | 71 | Colorectal | Metastatic | 63.66 | 30 |
| 32 | F | 71 | Colorectal | Metastatic | 59.23 | 18 |
| 33 | M | 71 | Colorectal | Metastatic | 75.39 | 12.3 |
| 34 | M | 71 | Colorectal | Metastatic | 61.40 | 17 |
| 35 | F | 71 | Colorectal | Metastatic | 30.20 | 2.6 |
| 36 | F | 71 | Colorectal | Metastatic | 35.84 | 0.4 |
| 37 | M | 72 | Colorectal | Metastatic | 71.03 | 11.3 |

| | | | | | | |
|---|---|---|---|---|---|---|
| 38 | M | 73 | Colorectal | Metastatic | 68.25 | 13.4 |
| 39 | M | 73 | Colorectal | Metastatic | 58.16 | 31 |
| 40 | M | 73 | Colorectal | Metastatic | 58.70 | 21 |
| 41 | F | 73 | Colorectal | Metastatic | 34.26 | 17.3 |
| 42 | F | 74 | Colorectal | Metastatic | 48.25 | 7.8 |
| 43 | M | 74 | Colorectal | Metastatic | 67.53 | 23.7 |
| 44 | F | 74 | Colorectal | Metastatic | 64.30 | 4.3 |
| 45 | M | 75 | Colorectal | Metastatic | 76.41 | 23 |
| 46 | M | 75 | Colorectal | Metastatic | 49.19 | 16.5 |
| 47 | F | 75 | Colorectal | Metastatic | 59.32 | 14.5 |
| 48 | M | 76 | Colorectal | Metastatic | 64.80 | 23.7 |
| 49 | M | 76 | Colorectal | Metastatic | 19.14 | 1.8 |
| 50 | M | 77 | Colorectal | Metastatic | 55.69 | 29 |
| 51 | M | 77 | Colorectal | Metastatic | 68.35 | 35.5 |
| 52 | M | 77 | Colorectal | Metastatic | 20.23 | 1.1 |
| 53 | M | 78 | Colorectal | Metastatic | 57.40 | 28 |
| 54 | M | 79 | Colorectal | Metastatic | 32.32 | 17.3 |
| 55 | F | 79 | Colorectal | Metastatic | 64.69 | 24.5 |
| 56 | F | 79 | Colorectal | Metastatic | 53.00 | 18.7 |
| 57 | F | 79 | Colorectal | Metastatic | 76.00 | 3.3 |
| 58 | F | 80 | Colorectal | Metastatic | 55.59 | 9.5 |
| 59 | M | 80 | Colorectal | Metastatic | 60.01 | 21.7 |
| 60 | F | 81 | Colorectal | Metastatic | 45.62 | 11.6 |
| 61 | F | 81 | Colorectal | Metastatic | 63.19 | 29 |
| 62 | M | 81 | Colorectal | Metastatic | 60.21 | 16.3 |
| 63 | F | 83 | Colorectal | Metastatic | 63.36 | 2.3 |
| 64 | F | 84 | Colorectal | Metastatic | 60.91 | 9.3 |
| 65 | F | 85 | Colorectal | Metastatic | 60.02 | 15.8 |
| 66 | F | 85 | Colorectal | Metastatic | 60.05 | 23 |
| 67 | M | 88 | Colorectal | Metastatic | 61.01 | 6.7 |
| 68 | F | 89 | Colorectal | Metastatic | 22.31 | 1.1 |
| 69 | F | 92 | Colorectal | Metastatic | 65.56 | 16.7 |
| 70 | M | 92 | Colorectal | Metastatic | 68.27 | 22.7 |

*It is very unlikely to develop colorectal cancer at the age of 23. However, we assume this patient may have a heritable predisposition.

**Supplementary Table 12. Normal cfDNA samples extracted from the plasma of healthy individuals. *NA= Not Applicable**

| Sample Number | Gender | Age | Sample Type | Cancer Stage | Mean Relative Adsorption (%$i_r$) | Mean Relative Absorbance ($A_{520/658}$) |
|---|---|---|---|---|---|---|
| 1 | F | 54 | Normal | NA | 3.98 | 1.02 |
| 2 | F | 31 | Normal | NA | 9.30 | 1.22 |
| 3 | F | 57 | Normal | NA | 33.31 | 7.53 |
| 4 | F | 18 | Normal | NA | 27.89 | 5.50 |
| 5 | F | 27 | Normal | NA | 17.95 | 7.15 |
| 6 | F | 54 | Normal | NA | 18.01 | 5.05 |
| 7 | F | 21 | Normal | NA | 18.53 | 2.14 |
| 8 | F | 23 | Normal | NA | 29.98 | 3.99 |
| 9 | F | 18 | Normal | NA | 5.60 | 0.96 |
| 10 | F | 43 | Normal | NA | 30.90 | 7.67 |
| 11 | F | 41 | Normal | NA | 16.93 | 2.13 |
| 12 | F | 25 | Normal | NA | 2.37 | 1.09 |
| 13 | F | 34 | Normal | NA | 0.97 | 1.15 |
| 14 | F | 47 | Normal | NA | 3.11 | 1.05 |
| 15 | F | 23 | Normal | NA | 4.22 | 0.86 |
| 16 | F | 41 | Normal | NA | 5.43 | 0.95 |
| 17 | F | 25 | Normal | NA | 22.76 | 4.03 |
| 18 | F | 46 | Normal | NA | 1.31 | 2.04 |
| 19 | F | 35 | Normal | NA | 28.17 | 5.08 |
| 20 | F | 49 | Normal | NA | 0.52 | 1.21 |
| 21 | F | 26 | Normal | NA | 21.3 | 2.27 |
| 22 | F | 46 | Normal | NA | 29.71 | 8.40 |
| 23 | F | 38 | Normal | NA | 35.34 | 4.00 |
| 24 | F | 48 | Normal | NA | 21.8 | 3.30 |
| 25 | F | 55 | Normal | NA | 43.83 | 10.60 |
| 26 | F | 60 | Normal | NA | 49.51 | 12.60 |
| 27 | F | N/A | Normal | NA | 22.4 | 7.00 |
| 28 | F | N/A | Normal | NA | 10.54 | 1.70 |
| 29 | F | N/A | Normal | NA | 0.59 | 2.08 |
| 30 | F | N/A | Normal | NA | 5.1 | 0.63 |
| 31 | F | 40 | Normal | NA | 56.2 | 8.40 |
| 32 | M | 56 | Normal | NA | 51.1 | 12.40 |
| 33 | M | 58 | Normal | NA | 18.31 | 2.40 |
| 34 | F | 59 | Normal | NA | 17.94 | 3.70 |
| 35 | M | 63 | Normal | NA | 10.37 | 0.30 |
| 36 | M | 63 | Normal | NA | 46.09 | 16.50 |
| 37 | M | 65 | Normal | NA | 33.26 | 11.50 |

| | | | | | | |
|---|---|---|---|---|---|---|
| 38 | M | 65 | Normal | NA | 29.21 | 7.70 |
| 39 | M | 67 | Normal | NA | 25.81 | 16.20 |
| 40 | F | 70 | Normal | NA | 14.25 | 6.20 |
| 41 | M | 70 | Normal | NA | 58.24 | 0.70 |
| 42 | M | 75 | Normal | NA | 34.96 | 16.70 |
| 43 | M | 77 | Normal | NA | 56.84 | 31.00 |
| 44 | M | 77 | Normal | NA | 42.57 | 8.30 |
| 45 | M | 78 | Normal | NA | 35.65 | 12.70 |
